# Cytoreductive Surgery Combined with Hyperthermic Intraperitoneal Intraoperative Chemotherapy in the Treatment of Advanced Epithelial Ovarian Cancer

**DOI:** 10.1155/2012/358341

**Published:** 2012-02-09

**Authors:** Antonios-Apostolos K. Tentes, Stylianos Kakolyris, Dimitrios Kyziridis, Christina Karamveri

**Affiliations:** ^1^Surgical Department, Didimotichon General Hospital, 68300 Didimotichon, Greece; ^2^Department of Medical Oncology, Democritus University of Thrace, 68100 Alexandroupolis, Greece

## Abstract

*Background/Aims*. Intraperitoneal intraoperative hyperthermic chemotherapy (HIPEC) has been used in the treatment of ovarian cancer. The purpose of the study is to determine the efficacy of HIPEC after cytoreductive surgery in advanced ovarian cancer. *Patients/Methods*. From 2006 to 2010 patients with advanced ovarian cancer were enrolled in a prospective nonrandomized study to undergo cytoreductive surgery combined with HIPEC. Clinical and histopathological variables were correlated to hospital mortality, morbidity, survival, and recurrences. *Results*. The mean age of 43 women was 59.9 ± 13.8 (16–82) years. The hospital mortality and morbidity rate were 4.7% and 51.2%, respectively. Complete cytoreduction was possible in 69.8%. The overall 5-year survival rate was 54%. The prognostic indicators of survival were the extent of prior surgery (*P* = 0.048) and the extent of peritoneal dissemination (*P* = 0.011). The recurrence rate was 30.2%. *Conclusions*. Maximal cytoreductive surgery combined with HIPEC is a well-tolerated, feasible, and promising method of treatment in advanced ovarian cancer.

## 1. Introduction

Epithelial ovarian cancer is usually diagnosed when the tumor has already disseminated at the peritoneal surfaces. The standard treatment at this stage is cytoreductive surgery combined with systemic chemotherapy [1]. Although ovarian cancer is one of the most chemosensitive tumors and complete response is achieved in 80% [2], the majority of patients develop recurrence, and long-term survival is poor [3–5]. The most significant prognostic variable of survival has been shown to be the maximal diameter of the residual tumor [6].

Even if a complete cytoreduction has been performed with no macroscopically visible tumor, microscopic tumor will always remain at the peritoneal surfaces. A potentially therapeutic result is possible if the residual tumor is eradicated. Intraperitoneal chemotherapy is effective in eradicating cancer emboli with maximal diameter less than 2-3 mm.

In practice, hyperthermic intraperitoneal intraoperative chemotherapy (HIPEC) has been used in locally advanced epithelial ovarian cancer as an adjuvant treatment after cytoreductive surgery with promising results [7–11].

The purpose of the prospective nonrandomized study is to determine the efficacy of HIPEC after maximal cytoreductive surgery in women with locally advanced epithelial ovarian cancer.

## 2. Patients/Methods

Women with locally advanced epithelial ovarian cancer (both primary and recurrent) were enrolled from 2006 to 2010 for maximal cytoreductive surgery with standard peritonectomy procedures combined with HIPEC.

The diagnosis was established by physical examination, hematological-biochemical examinations, tumor markers (CEA, CA 19-9, CA-125), and abdominal and thoracic CT scan. The performance status, age, the extent of prior surgery, the extent and distribution of peritoneal dissemination, the tumor volume, the completeness of cytoreduction (CC score), the presence of ascites, and the presence of metastatic disease were assessed and correlated to survival, recurrences, sites of recurrence, morbidity, and hospital mortality.

The physical status of the patients was assessed using the Karnofsky performance scale.

The extent of prior surgery was assessed using prior surgery score (PSS) [12]. The score was defined as PSS-0 when no surgery had been performed for cancer, as PSS-1 when biopsy only or surgery in one abdominopelvic region had been performed, as PSS-2 when surgery in 2–5 regions had been performed, and as PSS-3 when surgery had been performed in more than 5 regions.

The extent and distribution of peritoneal dissemination was assessed by using the peritoneal cancer index (PCI). Two transverse and two sagittal planes divided the abdomen in 9 regions. The upper transverse plane was the lowest part of the costal margin and the lower plane the anterior superior iliac spine. The small bowel was assessed as a separate entity, divided into 4 segments (upper and lower jejunum, upper and lower ileum). The peritoneal cancer index was the sum of the tumor volume in each one of the 13 abdominopelvic regions. The tumor volume was assessed as small if the largest tumor nodules were <0.5 cm in their largest diameter and as large if the nodules were >0.5 cm [12].

The completeness of cytoreduction was indicated by CC-0 to CC-3. A CC-0 indicated that no visible tumor had been left behind after surgery. A CC-1 indicated that the residual tumor was <0.25 cm in its largest diameter. If after cytoreductive surgery tumor >0.25 cm and <2.5 cm was left behind, it was indicated as CC-2 surgery, and when the largest diameter of the residual tumor was >2.5 cm this was indicated by CC-3 surgery. Only CC-0 operations were considered complete cytoreductions [12].

The presence of metastatic disease to remote lymph nodes that had no anatomic relationship to the primary site was considered as distant metastasis.

During the immediate postoperative period all patients were assisted in an intensive care unit for 24 hours. If early postoperative intraperitoneal chemotherapy (EPIC) was used then the patients were assisted for 5 days in the ICU. Chemotherapy toxicity was scored using the WHO criteria. Treatment-related morbidity was classified as grade 1: uncomplicated patient, grade 2: minor complications, grade 3: major complications requiring intervention (ICU readmission or reoperation), and grade 4: in-hospital mortality.

The protocol was approved by the Ethical Committee of the hospital, and an informed consent was signed by all patients.

Patients with: (a) acceptable physical status (Karnofsky performance status >50%), (b) normal liver and renal function, (c) normal hematological profile, and (d) no evidence of other malignancy or at risk for recurrence, except for basal cell carcinoma or in situ cervix cancer properly treated, were considered eligible for maximal cytoreductive surgery and HIPEC.

### 2.1. Treatments

The patients underwent surgery with the intention of performing a complete cytoreduction. The standard peritonectomy procedures used for maximal cytoreduction of the tumor volume were pelvic peritonectomy, greater omentectomy with or without splenectomy, lesser omentectomy, right and left subdiaphragmatic peritonectomy, cholecystectomy with resection of the omental bursa, and parietal peritonectomy. Resection of other organs, small and/or large bowel, and stomach was performed if necessary for achieving complete cytoreduction.

After the resection of the tumor and before the reconstruction of the gastrointestinal tract HIPEC was performed using the Coliseum technique [13] for 90 min if cisplatin (50 mg/m^2^) and doxorubicin (15 mg/m^2^) were instilled and for 60 min if gemcitabine (1000 mg/m^2^) was instilled at 42.5–43°C. Gemcitabine was used for platinum-resistant women. HIPEC was performed via a circuit of 4 drains (2 inflow and 2 outflow) that were connected to an extracorporeal sterile circuit in which a 3 lit perfusate was circulated by two peristaltic pumps (one inflow and one outflow) at a flow rate of 2 lit/min. The sterile circuit was heated by a thermal exchanger connected to the heating circuit. Systemic chemotherapy was used in those patients that underwent CC-1 or CC-2 surgery or those that had systemic or recurrent disease. Platinum-resistant patients were considered those women that did not respond or developed recurrence in less than 6 months after initial systemic chemotherapy.

### 2.2. Followup

The patients were followed up every 4 months during the first year after surgery and every 6 months later with physical examination, hematological-biochemical examinations, tumor markers (CEA, CA-125), and CT abdominal scan. The recurrences and the sites of recurrence were recorded.

### 2.3. Statistical Analysis

Statistical analysis was made using the SPSS (Statistical Package for Social Sciences). The proportions of patients with a given characteristic were compared by chi-square analysis or by Pearson's test. Differences in the means of continuous measurements were tested by the Student's *t*-test. The survival curves were obtained using the Kaplan-Meier method, and the comparison of curves was calculated using the log-rank test. Cox regression analysis made possible multiple analysis of survival. Logistic regression analysis made possible multiple analysis of recurrence and morbidity. A two-tailed *P* value <0.05 was considered statistically significant.

## 3. Results

From 2006 to 2010, 43 women with primary or recurrent ovarian cancer were enrolled in the study and underwent maximal cytoreductive surgery and HIPEC. The mean age of the patients was 59.9 ± 13.8 (16–82) years. The characteristics of the patients are summarized in Table 1. Twenty patients (46.5%) had recurrent ovarian cancer and underwent secondary cytoreduction. The mean PCI was 15.05 (3–33). Extensive peritoneal dissemination was found in 20 (46.5%) women, and their intraoperative PCI was calculated >15. The performed peritonectomy procedures are listed in Table 2. In 4 patients (9.3%) lymph nodal involvement was found in remote sites that had no anatomical relationship to the primary site. Despite the extent of peritoneal dissemination CC-0 surgery was possible in 30 cases (69.8%). Five patients with recurrent disease received gemcitabine during HIPEC because they were considered to be platinum resistant, and 15 patients received cisplatinum + doxorubicin.

### 3.1. Hospital Morbidity and Mortality

Grade 1 morbidity was recorded in 21 patients (48.8%). Grade 2 morbidity was recorded in 16 patients (35.2%) that had pleural effusion, neutropenia grade II that did not require medical treatment, pneumonitis, fistulas, and wound infection, grade 3 morbidity was recorded in 4 patients (9.3%) that had enterocutaneous fistulas, and grade 4 morbidity in 2 patients (4.7%) with anastomotic failure that developed sepsis (Table 3).

### 3.2. Histopathology

Histopathology revealed serous adenocarcinomas in 25 cases (58.1%), cystadenocarcinomas in 8 cases (18.6%), endometrioid in 6 cases (13.9%), and clear-cell carcinomas in 4 cases (9.4%).

### 3.3. Survival

**Figure 1:**
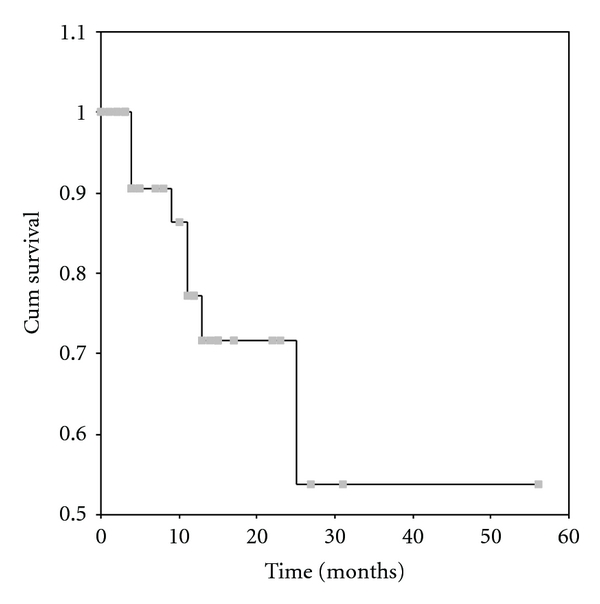
Overall 5-year survival rate.

**Figure 2:**
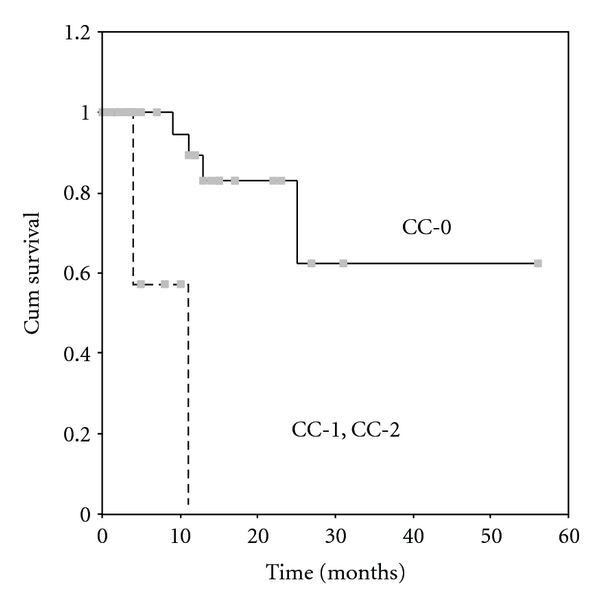
Survival according to CC score. The continuous line is for patients with CC-O and the dotted line is for patients with CC-1, CC-2 (*P* = 0.0001).

**Figure 3:**
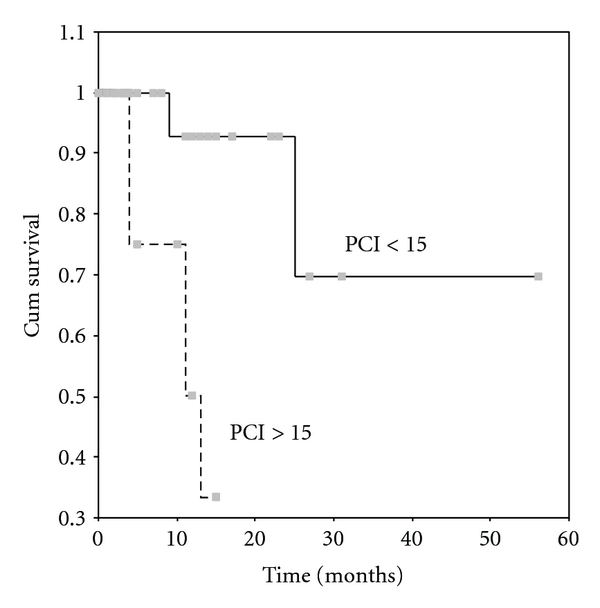
Survival according to PCI. The continuous line is for patients with PCI < 15 and the dotted line for patients with PCI > 15 (*P* = 0.0022).

**Figure 4:**
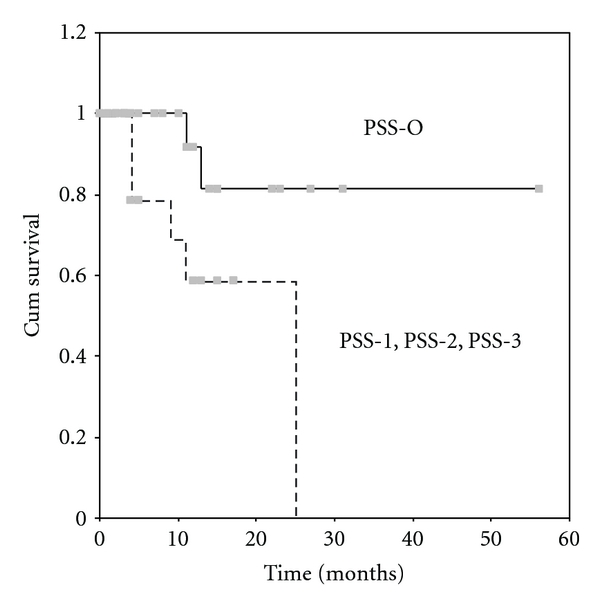
Survival according to PSS. The continuous line is for patients with PSS-0 and the dotted line for patients with PSS-1, PSS-2, and PSS-3 (*P* = 0.0265).

The overall 5-year survival rate was 54% (Figure 1). The mean survival was 37 ± 6 months. By univariate analysis it was found that the completeness of cytoreduction (*P* = 0.0001), the PCI (*P* = 0.0022), the PSS (*P* = 0.0265), the presence of ascites (*P* = 0.0476), and the use of systemic chemotherapy (*P* = 0.0383) were related to survival. 5-year survival rate for patients with complete cytoreduction was 62.5% (Figure 2), for those with a PCI < 15, 70% (Figure 3), and for those with a PSS-0, 82.5% (Figure 4).

 By multivariate analysis it was found that the prognostic indicators of survival were the PSS (HR = 5.844, *P* = 0.048, 95% CI = 1.017–33.588) and the PCI (HR = 20.425, *P* = 0.011, 95% CI = 1.975–211.22).

### 3.4. Followup

No patient was lost during followup. During followup 13 patients (30.2%) developed recurrence. The recurrence was distant in 5 patients and locoregional in 8. Of the 43 patients, 28 (65.1%) are alive without evidence of disease, 3 patients (7.1%) died for reasons unrelated to disease, 8 patients (18.6%) died because of recurrence, and 2 patients (4.6%) are alive with recurrence. By univariate analysis the recurrence was found to be related to pathological values of CA-125 (*P* = 0.022). No other variable was found to be related to the development of recurrence. The patients that received cisplatin + doxorubicin were not offered longer survival compared to those that received gemcitabine during HIPEC.

## 4. Discussion

The survival rate of epithelial ovarian cancer has improved because the tumor is one of the few most chemosensitive to platinum derivatives [2]. However, the long-term survival has still been poor and has not exceeded 20% [3–5]. New promising treatment strategies implemented the last decade give hope that survival will be improved.

The most powerful tool in the treatment of ovarian cancer with peritoneal dissemination is cytoreductive surgery. Complete (CC-0) or near-complete (CC-1) cytoreduction is feasible in more than 75% of the cases [14] if maximal cytoreductive surgery with standard peritonectomy procedures is used [15]. Extensive peritoneal carcinomatosis in ovarian cancer is an unfavorable prognostic indicator. However, patients with high values of PCI are not necessarily excluded from surgical intervention. The PCI can be approximately calculated preoperatively by CT abdominal scanning. There are limitations in the accuracy and specificity of the preoperative evaluation of the PCI using the CT scan. Tiny nodules at the peritoneal surfaces of the bowel are rarely depicted at the CT scan, and the preoperative calculation of the PCI is not always accurate. The caveat for complete cytoreduction is the extent of dissemination at the peritoneal surfaces of the bowel. This is the reason why 8 subtotal colectomies were performed in the present study. This aggressive method has been successfully used for the treatment of mucinous peritoneal carcinomatosis from nongynecologic cancer [16]. Therefore, high rate of complete (69.8%) or near-complete cytoreduction (25.6%) was possible although in 20 patients the PCI was >15 which is in agreement with other reports for patients with primary or recurrent ovarian cancer [7–9, 11, 14, 17, 18].

One of the most significant variables of survival is the extent of prior surgery. It has not been given much attention and only in one study that the PSS has been reported as a prognostic variable of survival [7]. The extent of prior surgery is probably related to the extent of tumor cell implantation at the peritoneal surfaces of the abdomen. It may also imply that the most significant variable for long-term survival is the first cytoreductive operation. In the present study it has been found that the initially diagnosed patients (PSS-0) have 82.5% 5-year survival rate. The extent of prior surgery and the extent of peritoneal carcinomatosis have also been identified as prognostic variables of survival. Paradoxically the completeness of cytoreduction has not been identified as a prognostic indicator although it has been found to be strongly related to survival [1, 6, 7, 9, 11].

Intraperitoneal chemotherapy has been documented as the standard treatment of peritoneal malignancy from nongynecologic cancer [19–23]. Intraperitoneal chemotherapy has also been used in ovarian cancer [24] and compared to intravenous chemotherapy has shown to improve survival in patients who had undergone in the past optimal cytoreductive surgery.

The method has been performed with the use of the open abdominal technique (Coliseum technique) that enables the uniform distribution of the heat and the cytostatic drugs in the abdominal cavity. In addition, during perfusion the surgeon has the advantage to surgically eradicate small nodules located at the mesentery of the small bowel and as a consequence to shorten the operative time.

Severe morbidity (grade 3 and 4) has been recorded in 6 patients (14%). It is obvious that the most severe complication is the anastomotic failure. Anastomotic failure has been reported in other series as the most frequent complication [8, 9, 25]. Cisplatin has been incriminated to impair anastomotic healing in animal studies [26] in contrast to local hyperthermia that has not [27]. As a consequence, the failures may be attributed either to cisplatin or to the immediate restoration of the gastrointestinal tract after low-anterior resection particularly in those cases with preoperative partial intestinal obstruction. The importance of intestinal obstruction and the avoidance of immediate restoration of the gastrointestinal tract has been emphasized [9] resulting in significant decrease of anastomotic failures [28]. Therefore a protective colostomy seems to be a reasonable solution. Other severe complications as intra-abdominal abscess or sepsis or postoperative bleeding are infrequent [8, 9, 25].

Severe side effects attributed to HIPEC have not been recorded. Grade 3/4 toxicity is rare and does not exceed 5% [25]. Only 3 patients have had grade II transient neutropenia requiring no specific treatment.

## 5. Conclusions

 Maximal cytoreductive surgery with standard peritonectomy procedures combined with intraperitoneal chemotherapy is a well-tolerated and feasible method for treatment of advanced epithelial ovarian cancer. It appears to improve long-term survival securing that complete or near complete cytoreduction is possible in the vast majority as well as the eradication of the microscopic residual tumor.

## Figures and Tables

**Table 1:** Characteristics of the patients.

	Number of patients	%
Performance status		
90–100%	34	79.1
70–80%	8	18.6
50–60%	1	2.3
Tumor volume		
Large volume	34	79.1
Small volume	9	20.9
PSS		
PSS-0	23	53.5
PSS-1	4	9.3
PSS-2	8	18.6
PSS-3	8	18.6
PCI		
PCI < 15	23	53.5
PCI > 15	20	46.5
CC		
CC-0	30	69.8
CC-1	11	25.6
CC-2	2	4.7
Ascites	27	62.8
Remote lymph nodes	4	9.3
Systemic chemotherapy	23	53.5
Morbidity	22	51.2
Hospital mortality	2	4.7
Recurrence	13	30.2

**Table 2:** Peritonectomy procedures.

Peritonectomy	Number of procedures	%
Right subdiaphragmatic	21	8.7
Left subdiaphragmatic	13	5.4
Greater omentectomy	37	15.4
Lesser omentectomy	12	5
Splenectomy	20	8.3
Pelvic peritonectomy	43	17.8
Cholecystectomy + resection of the omental bursa	23	9.5
Right parietal	20	8.3
Left parietal	20	8.3
Segmental intestinal resection	12	5
Right colectomy	6	2.5
Subtotal colectomy	8	3.4
Abdominopelvic lymph node resection	2	0.8
Antrectomy	4	1.6

**Table 3:** Complications.

Complication	Number of patients	%
Grade I	21	48.8
Grade II		
Pleural effusion	2	4.7
Wound infection	7	14.2
Neutropenia grade II	3	6.9
Pneumonitis	2	4.7
Enterocutaneous fistulas	2	4.7
Grade III	4	9.3
Enterocutaneous fistulas		
Grade IV	2	4.7
Anastomotic failure		
